# In situ 3D topographic and shape analysis by synchrotron radiation X-ray microtomography for crystal form identification in polymorphic mixtures

**DOI:** 10.1038/srep24763

**Published:** 2016-04-21

**Authors:** Xian-Zhen Yin, Ti-Qiao Xiao, Ashwini Nangia, Shuo Yang, Xiao-Long Lu, Hai-Yan Li, Qun Shao, You He, Peter York, Ji-Wen Zhang

**Affiliations:** 1 Center for Drug Delivery System, Shanghai Institute of Materia Medica, Chinese Academy of Sciences, Shanghai 201210, China; 2 Institute of Pharmaceutical Innovation, University of Bradford, Bradford, West Yorkshire BD7 1DP, United Kingdom; 3 Shanghai Synchrotron Radiation Facility, Shanghai Institute of Applied Physics, Chinese Academy of Sciences, Shanghai 201204, China; 4 School of Chemistry, University of Hyderabad, Hyderabad 500046, India

## Abstract

Polymorphism denotes the existence of more than one crystal structure of a substance, and great practical and theoretical interest for the chemical and pharmaceutical industries. In many cases, it is challenging to produce a pure crystal form and establish a sensitive detection method for the identification of crystal form in a mixture of polymorphs. In this study, an accurate and sensitive method based on synchrotron radiation X-ray computed microtomography (SR-μCT) was devised to identify the polymorphs of clopidogrel bisulphate (CLP). After 3D reconstruction, crystal particles were extracted and dozens of structural parameters were calculated. Whilst, the particle shapes of the two crystal forms were all irregular, the surface of CLP II was found to be rougher than CLP I. In order to classify the crystal form based on the quantitative morphological property of particles, Volume Bias Percentage based on Surface Smoothing (VBP) was defined and a new method based on VBP was successfully developed, with a total matching rate of 99.91% for 4544 particles and a lowest detectable limit of 1%. More important for the mixtures in solid pharmaceutical formulations, the interference of excipients can be avoided, a feature cannot achieved by other available analytical methods.

Polymorphism is the ability of a solid material to exist in more than one crystal structure, which can potentially be found in many crystalline materials including polymers, minerals, and metals. Polymorphism is of great theoretical and practical interest for the pharmaceutical industries, since different polymorphs of the same compound exhibit diverse physicochemical properties of solubility, dissolution rate, stability, and mechanical properties, thereby influencing the bioavailability and therapeutic efficiency of the formulated drug products^1^,^2^. Many polymorphic drug substances approved by the regulatory authorities for one specific polymorph of the compound^3^. However, individual polymorphs may convert to another during manufacturing process and storage, particularly when a metastable form is used and a resulting more stable polymorph is likely to exhibit a lower dissolution rate. Since an amorphous form is thermodynamically less stable than the crystalline form, spontaneous crystallization from an amorphous drug substance may also occur^4^,^5^. Therefore, the investigation of crystal polymorphism of active pharmaceutical ingredients (APIs) is of increasing importance to the pharmaceutical industry, either in the early stage of drug discovery research or the development and manufacture of a drug delivery system.

Several techniques can be applied to identify and characterize solid-state forms and polymorphic composition of APIs in raw materials and dosage forms, such as X-ray diffraction (XRD), thermal methods (including differential scanning calorimetry (DSC), thermal gravimetric analysis (TGA) and isothermal microcalorimetry (IMC)), vibrational spectroscopy (including mid-infrared (IR), near-infrared (NIR), Raman, and terahertz pulsed spectroscopy (TPS)), solid state NMR (SS-NMR), atomic force microscopy (AFM), optical and electron microscopy^6^,^7^,^8^,^9^. Whilst powder X-ray diffraction (PXRD) has increasingly been regarded as the definitive test for the crystallographic identification of polymorphs, it can only be used to quantify non-crystalline or second component crystalline material down to levels of 5%^1^. In addition, its use for quantitative analysis is often marred for samples with complex scattering patterns, since the isolation of the diffraction peaks of the APIs and excipients peaks may be difficult and some low intensity peaks may exist in the background because of the effect of residual solvent in the sample, factors which may interfere with the interpretation of results^1^.

For clopidogrel bisulphate (CLP), six polymorphs are known, but only two (Form I and form II) are used as therapeutic agents. Form II as well as several solvate forms and amorphous form of the substance are patented, but Form I is now out of patent control and available for generic product development and marketing^10^,^11^. As clopidogrel bisulphate polymorphs comprise an enantiotropic system, and Form II is the thermodynamically more stable form at room temperature^12^, there is a potential for the occurrence of Form II in Form I both in the production steps and during the storage period. Thus, a suitable analytical technique for the detection and quantification of low levels of the stable form II in the metastable Form I material is needed. A recent publication has reported on the quantitative analysis of clopidogrel bisulphate polymorphs by X-ray powder diffraction^10^. The authors used whole powder pattern decomposition as well as classical direct methods for quantization in the range of 10–80% Form I in Form II. The limit of detection using both methods is in the range of 1–2% of phase content in the mixture. A previous study utilized transmission FT-IR spectroscopy for the quantitative measurement of clopidogrel bisulphate polymorphs^11^, by using unique peaks of the forms in the analytical range of 10–90% Form I in Form II. Low levels of Form II in Form I are undetectable because characteristic bands of Form II are not visible in the IR spectra of mixture below 30%. Recently, vibrational spectroscopic methods have been developed for quantitative analysis of Form II of clopidogrel bisulphate in Form I and Form II polymorphic mixtures. Results show that both IR and Raman spectroscopy combined with chemometrics are suitable to quantify low levels of Form II in Form I, down to 2 and 3%, respectively, with less than 1% limit of detection^12^.

However, it is still a great challenge to identify quantitatively the polymorphs in multi-component mixtures, or with binary mixtures in which the crystalline form of interest is present at a low level. The microscopic methods based on the image acquisition followed by a suitable image-analysis scheme to extract morphological details may be useful tools to quantify the trace amount of polymorphs by morphology. These approaches are especially powerful for the characterization of minor phases in mixtures and, provided that a suitable crystallite can be located, the analysis can be performed on exceptionally small amounts of material. The particular value of these methods lies in the possibility it offers for combining imaging, diffraction and spectroscopic data from the same sample, with the potential for simultaneous acquisition of different data types. For example, in scanning mode, direct imaging of the crystal lattice can be coupled with spectroscopic analysis to yield chemical information at the unit cell level; in imaging mode, the technique is routinely used for the identification and characterization of defective structures; and in diffraction mode, detailed investigations of both symmetry and crystal structure are possible^13^,^14^.

X-ray computed tomography (μCT), a powerful non-invasive investigation technique applied for observing the three-dimensional structure of various objects, has great potential for providing fundamental understanding of the properties of crystalline solids and analyzing crystal morphology of APIs within the solid preparations such as powders, tablets and capsules. In contrast to conventional techniques, the μCT technique also allows non-invasive, visualization of internal microstructural details at micron or submicron resolution^15^,^16^. Compared with the conventional X-ray computed microtomography, the synchrotron radiation light source can provide ten thousand times higher photon flux in parallel beam morphology. Moreover, synchrotron radiation X-ray computed microtomography (SR-μCT) permits the rapid acquisition of data with high intensity and strong collimation via a high performance detector^16^,^17^,^18^.

The present study employed SR-μCT to devise and develop an accurate and sensitive method for identification and quantification of polymorphs, using forms I and II of CLP crystal particles as a model. The SR-μCT was firstly employed to visualize the morphology and to identify qualitatively CLP I and CLP II. The steric parameters of CLP polymorph particles, such as size, sphericity and surface properties were calculated. Then a new parameter, VBP was derived which eliminated interference from any other excipients (inactive substances used for the formulation of pharmaceutical preparations) to achieve the quantitative identification of polymorphs with an *in-situ* 3D view.

## Results and Discussion

### CLP polymorphs characterization

**Figure 1:**
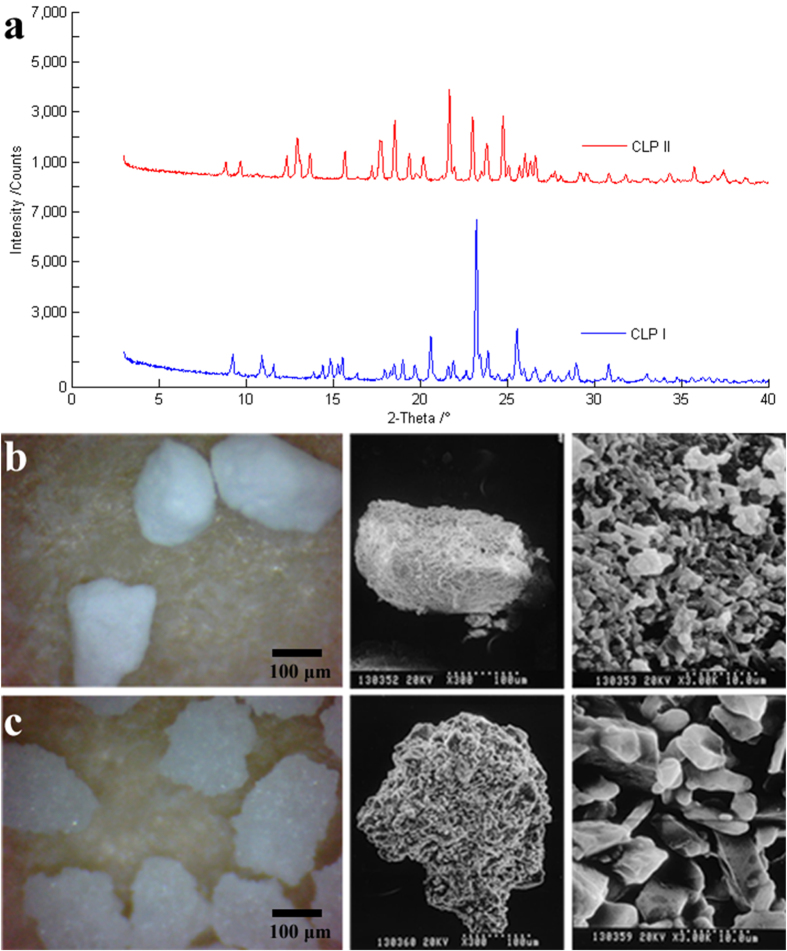
PXRD and morphology of CLP I and CLP II crystal particles. (**a**) PXRD, (**b**) optical microscopy and SEM morphology of CLP I, (**c**) optical microscopy and SEM morphology of CLP II.

The PXRD profiles of CLP I and II are shown in Fig. 1, and these are in good agreement with reported literatures^11^,^19^.

The optical microscopy and SEM images in Fig. 1 indicate that particles of the two polymorphs are all polycrystalline rather than individual larger single crystals, but the primary particles within the polycrystalline assemblies of CLP I particles are smaller and more spherical, in contrast with the larger columnar single primary crystalline units of CLP II which form an irregular and rough surface for the polycrystalline assembly.

### Quantitative characterization of the polymorphs

**Figure 2:**
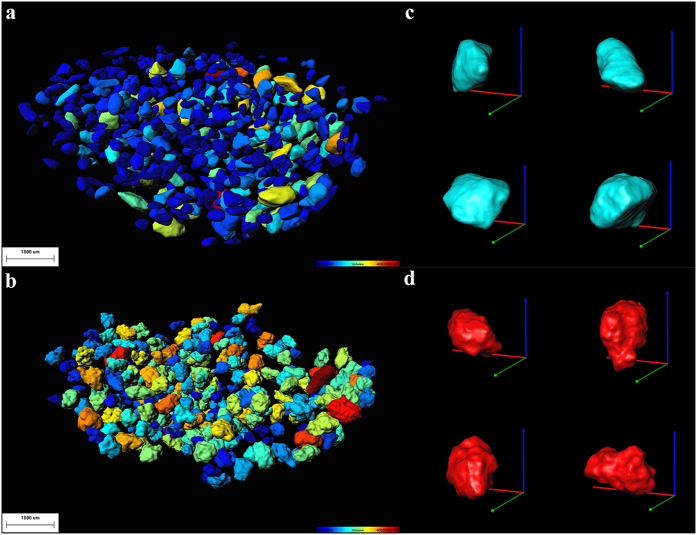
3D images of individual CLP I and CLP II microcrystalline particles in capsule after extraction and construction: (**a**) Particles of CLP I and (**b**) particles of CLP II. Morphologies of randomly selected four particles (**c**) particles of CLP I and (**d**) particles of CLP II.

Based on the analysis of gray values, all of the crystal particles in the sample were extracted by the gray level segmentation. Then the highly resolved tomographic images of CLP I and CLP II with high quality phase contrast were derived for each single particle after 3D reconstruction as shown in Fig. 2. Both samples had hundreds of individual microcrystalline particles, which were colored with pseudo-color according to the volumes of particles. Then, four particles of each crystal form were randomly selected to show the morphologies and spatial information (Fig. 2). As can be seen, the morphologies of the CLP I and CLP II differ from each other kind slightly. The particle shapes of the two crystal forms were all irregular but the surface of CLP II exhibited a greater degree of topographical roughness than CLP I, a finding which is in good agreement with that showed in the optical microscopy and SEM images.

**Figure 3:**
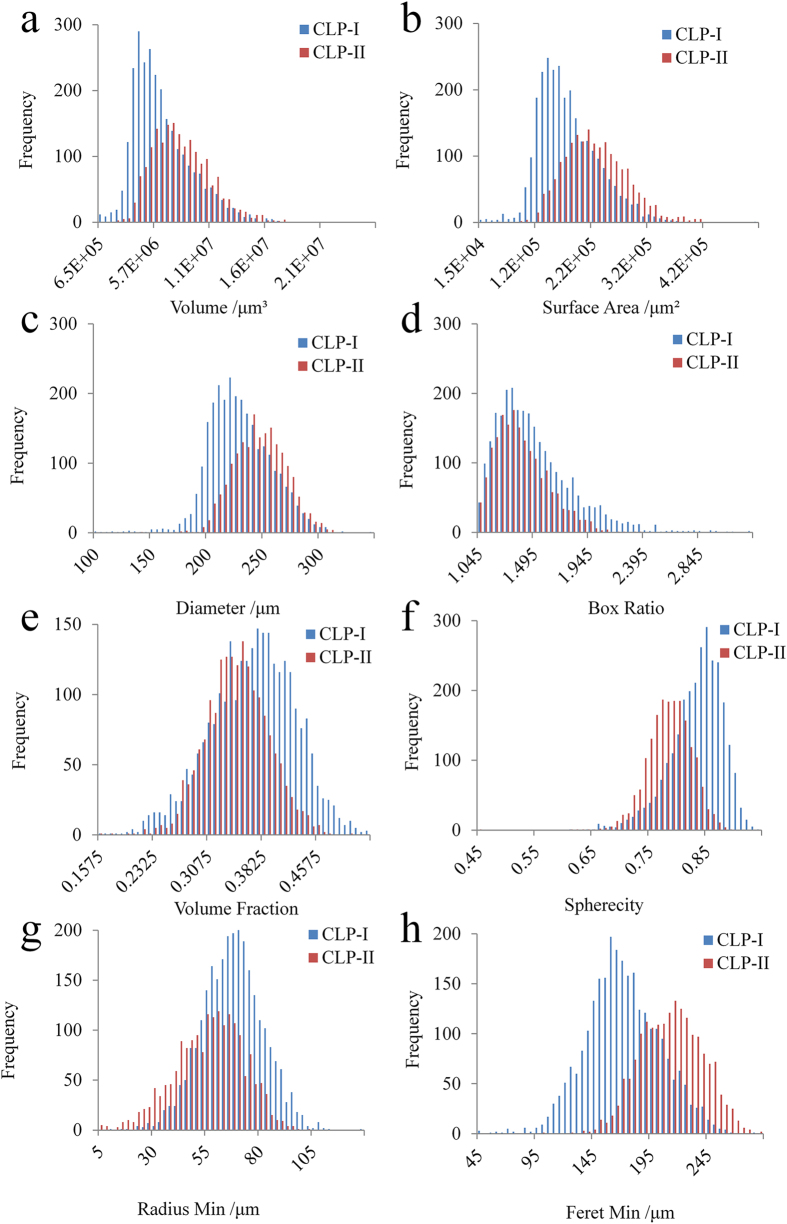
Frequency distribution profiles of individual CLP I and CLP II microcrystalline particles. (**a**) Volume, (**b**) Surface area, (**c**) Diameter, (**d**) Box ratio, (**e**) Volume fraction, (**f**) Sphericity, (**g**) Radius min, (**h**) Feret min (n = 4544).

Based on characteristic data of single particle, the individual particles cluster of CLP I and CLP II are described by means of the frequency distribution of volume, surface area, diameter, box ratio, volume fraction, sphericity, radius min and Ferret min, as shown in Fig. 3. In comparison with the CLP I particles, the diameter distribution shows that the particle cluster of CLP II has a larger average diameter, although both clusters were all fractionated with the same standard sieve (CLP I covering from 150 μm to 400 μm, CLP II covering from 200 μm to 400 μm). This finding is likely to be caused by the difference in morphological properties between the two crystal forms, as can be seen from Fig. 2. The CLP I microcrystalline particles are all oblate objects with relatively smooth surfaces in contrast to those of CLP II which are all much more irregular, rough and complex. The particle cluster of CLP I exhibits a wider distribution range in volume, surface area and diameter. The distribution of box ratio indicates that the microcrystalline particles of CLP II are thinner and flatter, and the particle cluster of CLP I shows low values in volume fraction and sphericity as the shape of the particles is more regular. The CLP I particles are narrow and flat in shape with a relatively smooth surface. In comparison with the particle cluster of CLP I, the CLP II shows a smaller radius min and larger Feret min indicating that the shapes of particles are more irregular and have deep voids and pores present on the surface.

### Quantitative analysis of the surface morphology

**Figure 4:**
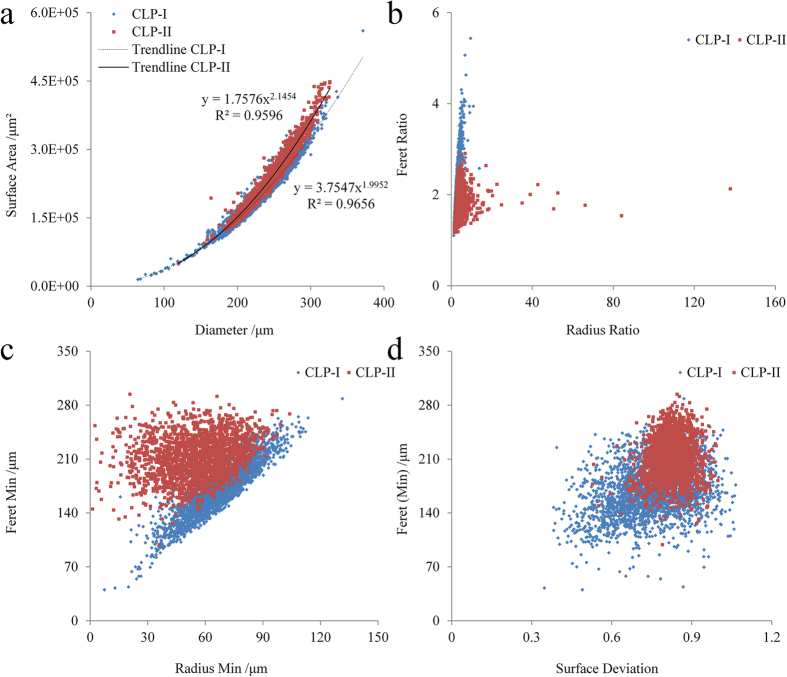
Relations between two different parameters. (**a**) Relation between diameter and surface area, (**b**) Radius ratio and Ferret ratio, (**c**) Radius min and Ferret min, (**d**) Surface deviation and ferret min (n = 4544).

In order to establish a quantitative method to identify CLP I and II, relationships between the 3D steric parameters have been investigated. As shown in Fig. 4a, the equivalent diameters and the surface area of particles and the trend lines of CLP I and CLP II show a standard cubic relationship, and a degree of differentiation. As the exponential index in the fitting equation for CLP I is 1.992 and close to 2, the integer for a perfect relationship between the diameter and surface area for a sphere. In contrast, the corresponding value for CLP II is 2.1454, higher than that for CLP I and larger than 2, demonstrating that the surface of CLP II is more complex and irregular.

The same tendency can also be seen from Fig. 4b–d, especially the correlation between the Feret min and the radius min. The correlation for CLP I shows a good linear relationship which indicates that the CLP I microcrystalline particles are regular with a flat facet, whilst on the other hand, the Feret min of CLP II particles has a significantly higher value than the radius min due to the coarser surface and pores distributed on the surface of particle. Furthermore, these findings obtained from the morphological parameters are consistent with the SEM results. However, data for CLP I and II in these correlations are all overlapping and require separation to achieve direct and quantitative polymorphic identification. Thus, new quantitative parameters and methods need to be created, developed and validated to characterize the roughness and pattern of surface.

### Crystal form classification based on surface morphology patterns

A crystal or crystalline solid is a solid material whose constituent atoms, molecules, or ions are arranged in an ordered pattern extending in all three spatial dimensions to form a periodic arrangement. In addition to their microscopic structure, large crystals are usually identifiable by their macroscopic geometrical shape, consisting of flat faces with specific, characteristic orientations.

An ideal crystal is a single crystal with a perfectly regular lattice in regular shapes, consisting of flat faces (also called facets) which are oriented in a specific way. In reality, a crystal particle’s external shape is determined by the crystal structure (which restricts the possible facet orientations), the specific crystal chemistry and bonding (which may favor some facet types over others), and the conditions under which the crystal formed. In practice and common use, individual particles are composed of a number of smaller crystals – a microcrystalline assembly - and are not regular in morphology. Therefore, we propose a hypothesis: that the surface morphology of typical particles composed of microcrystals has a high specificity which is different from other polymorphs of the same chemical compound. Thereby the shape pattern of the surface is closely related and determined by the crystal form as polymorphs frequently exhibit some difference in single crystal shape and the aggregation mode whereby single crystal form microcrystalline assemblies to form particles. It is proposed that these patterns can be used for the classification of the polymorph.

**Figure 5:**
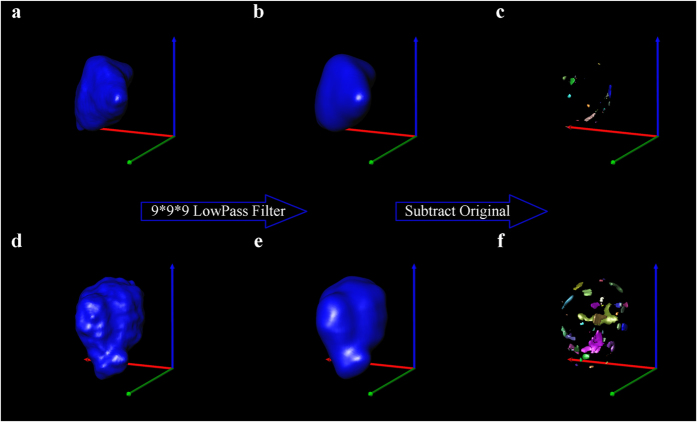
Quantitative characterization of the particles’ surface pattern: the original 3D model of the CLP I (**a**) and CLP II (**d**) particles, the 3D model of the CLP I (**b**) and CLP II (**e**) particles after smooth with 9^*^9^*^9 low pass filter algorithm, volume bias of the CLP I (**c**) and CLP II (**f**) particles.

In order to quantitatively classify the crystal form based on the morphological property of particles, a new method has been developed for the characterization of the pattern of the particles’ surfaces. At first, after the 3D model construction, every particle in the sample was extracted individually and moved into a sufficiently empty computing space; secondly, the original 3D model was processed by the low pass filter algorithm at the degree of 9 × 9 × 9 (the quantitative analysis of surface pattern indicates that the sizes of pores distributed on the surface were close to 9 × 9 × 9) for smoothing the surface to generate a new smoothed 3D model; then the smoothed model was sliced into a stack of 2D slices, and the original slice was subtracted from the new stack; finally, the residual part after subtraction was used to construct the deviation model. The sum volume of all objects in the model divided by the volume of original model was defined as the Volume Bias for the characterization of surface roughness (Fig. 5).


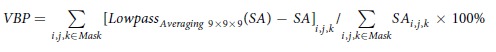


where

*VBP* is the Volume Bias Percentage based on Surface Smoothing.

*SA* is the original 3D matrix of crystal particle.


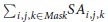
 is the volume of crystal particle above the threshold value.


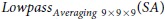
 is the lowpass filtering of 3D matrix of crystal particle with the averaging algorithm at the size of 9 × 9 × 9.

**Figure 6:**
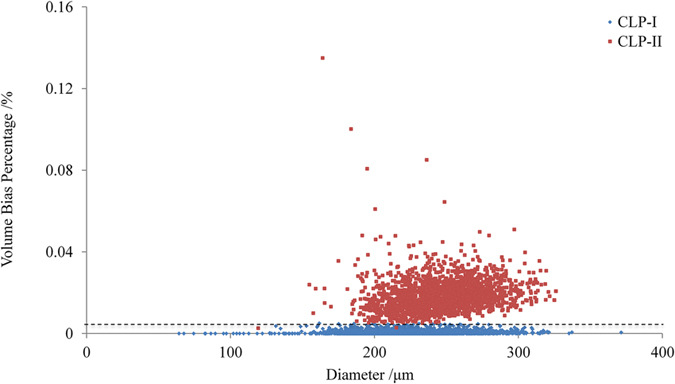
Volume bias analysis of 4544 CLP I (n = 2710) and CLP II (n = 1834) particles.

In the experiment, totally 4544 (include 2710 particles of CLP I and 1834 particles of CLP II) particles have been prepared and scanned, and the respective VBP values were calculated. As shown in Fig. 6, the VBP values clustered into two classes with values of CLP II generally higher than those of CLP I. The volume bias values of CLP I have a narrow distribution range, and most are close to 0. In contrast, the distribution of volume bias values of CLP II is wide and several particles have significantly high values. After analysis, the highest 1% of CLP I and the lowest 1% of CLP II (totally 45 particles include 27 CLP I and 18 CLP II) VBP values were used to calculate an average value. In this way the average value of 0.004915 has been obtained and was selected as the threshold value for the classification of two crystal forms. The overall accuracy is 99.91% (4 particles misidentified out of 4544) as there are 2 CLP I and 2 CLP II particles misidentified by the VBP value. As listed in the support information Table S1, the one by one matching analysis indicates the average accuracy was 99.82 ± 0.21% at 9 different polymorphic mix ratios. The lowest detectable limit of 1% has been observed.

## Conclusion

This research has proved that SR-μCT is a powerful non-invasive and quantitative technique that can be used for investigation of microcrystalline particles at high resolution. The structure of every single microcrystalline particle can be visualized from the 3D X-ray CT images. With the N-in-One analysis, from only one CT scan, the 3D morphological information of hundreds even thousands of crystal particles can be obtained; also a wide range of 3D quantitative parameters can be calculated. Generally, the solid form morphology of a chemical compound is defined in terms of its internal structure. Beside the crystal form, crystal habit described the shapes and aggregates that a certain compound is likely to form. Once the crystallization condition is fixed, the external appearance could be predictively determined. The morphology is also an important character for the identification of crystal form, whereas the challenge is the quantitative characterization of 3D morphology of crystalline particles. In this research, the concept of volume bias percentage (VBP) has been proposed for the quantitative characterization of the surface roughness and the surface morphology pattern. Thus a new method has been developed for the quantitative identification of crystal form of clopidogrel crystal at a high accuracy level (above 99.91%). Importantly, interference of excipients, which is commonly found, can be avoided a fact which demonstrates an advantage of this technique over other polymorph identification methods. It is of interest that this research has demonstrated the identification of crystal form based on the quantitative 3D morphology.

## Methods

### Materials

CLP I and CLP II, were provided by Shenyang Pharmaceutical University (Shenyang, China) with the purity of 99.9%. PVP/VA (Plasdone® S630) purchased from Shanghai Chineway Pharmaceutical Tech Co., Ltd. (Shanghai, China) was used as diluents due to its stability, good flowability and low X-Ray absorption coefficient to separate the CLP particles from each other. Both the CLP I and CLP II crystal forms were fractionated by sieving to obtain a particle size between 180–280 μm, and all other materials were fractionated by sieving to obtain a particle size between 70–150 μm. The gelatin capsule shell of size 2^#^ was provided by Xinchang Hechang Capsule Equipment Co. Ltd. (Zhejiang, China) as the container of powder sample. CLP particles and excipients were mixed thoroughly and filled in the capsules. The CT scans were carried out at Shanghai Synchrotron Radiation Facility (SSRF) at BL13W beam line.

### CLP polymorphs characterization

The PXRD was performed to verify the crystal form of the two polymorphs following pulverization (<5 μm) and homogenization. PXRD scans were acquired using a Bruker D8 Advance (Siemens) powder diffractometer equipped with a 2.2 kW sealed Cu X-ray source, a graphite monochromator to filter out the Cu K β radiation, and a NaI (Tl) scintillation detector. The scans were performed between 3° and 40° 2θ with a 0.01° step size and a counting time of 0.1 s per step.

The morphological information of CLP particles was also acquired using optical microscopy and SEM. The optical microscopy images were acquired with magnification at 200× (1280 × 960 pixels and pixel size was 0.450 μm), and for the SEM test all polymorph samples were scanned at 300× (800 × 900 pixels and pixel size was 0.450 μm) and 3000× (800 × 900 pixels and pixel size was 0.045 μm) respectively to capture the full surface morphological details of particles.

### Sample preparation

In order to extract and characterize every particle individually from a mixture sample with hundreds of particles, PVP/VA was introduced as typical and widely used excipients and diluents. The stable, smaller size, good flowability, and X-ray absorption contrast of the diluents facilitate the CLP particle extraction during image processing.

CLP I and CLP II (−60 mesh~ +80 mesh, size between 180–280 μm) 5 mg were weighed and filled into the capsule with 10 mg of PVP/VA respectively. In order to ensure the CLP crystals and excipients were blended thoroughly, the capsule was rotated manually and horizontally at a speed of 30 rotations per minute (rpm) for 30 seconds. The capsule was then fixed at the center of the stage positioned in the synchrotron beam line, then the heights of the experimental acquisition window were adjusted to cover the whole sample in the capsule and then the CT scan was carried out.

### SR-μCT scans and 3D reconstruction

SR-μCT tomographic images were acquired with beam line BL13W1 at SSRF. Samples were scanned with synchrotron radiation X-ray at 16.0 KeV. After penetration through the sample, the projections were magnified by diffraction-limited microscope optics (2× magnification). Then X-rays were converted into visible light by a Lu2SiO5: Ce scintillator (10 μm thickness) and digitized by a high-resolution 2048 pixel × 2048 pixel CCD camera (pco.2000, PCO AG, Kelheim, Germany). The pixel size was 3.7 μm, the exposure time was 2.0 s and the sample-to-detector distance was 12 cm. For each acquisition, 720 projection images over 180° were taken. Light field images (i.e. X-ray illumination on the beam-path without the sample) and dark-field images (i.e. X-ray illumination off) were also collected during each acquisition, for the correction of electronic noise and variations in the X-ray source brightness.

The projected images were reconstructed using the software developed by SSRF to perform a direct filtered back-projection algorithm^20^. In order to enhance the quality of reconstructed slices, the X-TRACT SSRF CWS x64 (Version 6.5, Commonwealth Scientific and Industrial Research Organization, Australia, http://www.ts-imaging.net/Default.aspx) was used for phase contrast extraction. The 3D rendered data were analyzed with commercially available software VGStudio Max (Version 2.1, Volume Graphics GmbH, Germany) and Image Pro Analyzer 3D (Version 7.0, Media Cybernetics, Inc., USA) to obtain qualitative and quantitative data respectively.

### Quantitative characterization and classification of the polymorphs

After segmentation, image slices were all converted into black and white images by removal of the background and noise. Then 3D ISO-Surface models were constructed with segmented slices. The surface level, surface range and the simplification parameters were adjusted to optimize the models. Then all of the CLP particles in the samples were extracted and characterized quantitatively. Finally, in order to classify CLP I and II quantitatively, several steric parameters were calculated and the overall surface patterns of the two polymorphs were presented by the new designed quantitative parameter, Volume Bias Percentage (VBP) based on Surface Smooth.

### Validation of the quantitative polymorph classification methods based on VBP

For the previously mentioned samples in the section of Sample Preparation, the sample of CLP II particles and excipient was transferred into the capsule with CLP I after the CT scans, and rotated to mix thoroughly and the capsule was positioned in the beam line as detailed above. Then, another CT scan was performed. In total 27 samples have been examined, and details are shown in Table 1.

After 3D reconstruction the samples were divided into 3 groups: group A (CLP-1, 4, 7, 10, 13, 16, 19, 22, and 25) with pure CLP I, group B (CLP-2, 5, 8, 11, 14, 17, 20, 23 and 26) with CLP II and group C (CLP-3, 6, 9, 12, 15, 18, 21, 24 and 27) with mixtures of polymorphs. For every sample, all particles were extracted and the Volume Bias Percentage based on Surface Smooth was calculated individually. Then the distribution of VBP values for group A and group B was compared, and threshold values for the classification of crystal forms were calculated. The number of misclassified particles was also considered to provide an estimate of the accuracy of method.

For samples in group C, particles in the polymorphs mixture were extracted one by one. After the classification of the crystal form with the VBP value, every particle was matching with the corresponding pure crystal form sample based on the surface morphological information and quantitative parameters such as volume, surface area and sphericity. By counting the number of mismatched particles, a method was developed to determine the lowest detectable limit of the experimental procedure.

## Additional Information

**How to cite this article**: Yin, X.-Z. *et al*. In situ 3D topographic and shape analysis by synchrotron radiation X-ray microtomography for crystal form identification in polymorphic mixtures. *Sci. Rep.* **6**, 24763; doi: 10.1038/srep24763 (2016).

## Supplementary Material

Supplementary Information

## Figures and Tables

**Table 1:** Detailed information of samples.

Sample ID	CLP I (mg)	CLP II (mg)	Excipients (mg)	Description
CLP-1	2.50	0.00	10.0	Pure CLP I
CLP-2	0.00	2.50	10.0	Pure CLP II
CLP-3	2.50	2.50	20.0	CLP-1 + CLP-2
CLP-4	1.00	0.00	10.0	Pure CLP I
CLP-5	0.00	4.00	10.0	Pure CLP II
CLP-6	1.00	4.00	20.0	CLP-4 + CLP-5
CLP-7	4.00	0.00	10.0	Pure CLP I
CLP-8	0.00	1.00	10.0	Pure CLP II
CLP-9	4.00	1.00	20.0	CLP-7 + CLP-8
CLP-10	0.50	0.00	10.0	Pure CLP I
CLP-11	0.00	4.50	10.0	Pure CLP II
CLP-12	0.50	4.50	20.0	CLP-10 + CLP-11
CLP-13	4.50	0.00	10.0	Pure CLP I
CLP-14	0.00	0.50	10.0	Pure CLP II
CLP-15	4.50	0.50	20.0	CLP-13 + CLP-14
CLP-16	0.25	0.00	10.0	Pure CLP I
CLP-17	0.00	4.75	10.0	Pure CLP II
CLP-18	0.25	4.75	20.0	CLP-16 + CLP-17
CLP-19	4.75	0.00	10.0	Pure CLP I
CLP-20	0.00	0.25	10.0	Pure CLP II
CLP-21	4.75	0.25	20.0	CLP-19 + CLP-20
CLP-22	0.05	0.00	10.0	Pure CLP I
CLP-23	0.00	4.95	10.0	Pure CLP II
CLP-24	0.05	4.95	20.0	CLP-22 + CLP-23
CLP-25	4.95	0.00	10.0	Pure CLP I
CLP-26	0.00	0.05	10.0	Pure CLP II
CLP-27	4.95	0.05	20.0	CLP-25 + CLP-26
