# Supplementary material for: In situ 3D topographic and shape analysis by synchrotron radiation X-ray microtomography for crystal form identification in polymorphic mixtures
Source: Sci Rep. 2016 Apr 21;6:24763. doi: 10.1038/srep24763 (PMC4838872; doi:10.1038/srep24763)
Supplement: Supplementary Information [file srep24763-s1.doc]

**Title:**

In situ 3D topographic and shape analysis by synchrotron radiation X-ray microtomography for crystal form identification in polymorphic mixtures

**Authors:**

Xian-Zhen Yin^1,2^, Ti-Qiao Xiao^3^, Ashwini Nangia^4^, Shuo Yang^1^, Xiao-Long Lu^1^, Hai-Yan Li^1^, Qun Shao^2^, You He^3^, Peter York^2^ & Ji-Wen Zhang^1^

**Affiliations:**

^1^Center for Drug Delivery System, Shanghai Institute of Materia Medica, Chinese Academy of Sciences, Shanghai 201210, China; ^2^Institute of Pharmaceutical Innovation, University of Bradford, Bradford, West Yorkshire BD7 1DP, United Kingdom; ^3^Shanghai Synchrotron Radiation Facility, Shanghai Institute of Applied Physics, Chinese Academy of Sciences, Shanghai 201204, China; ^4^School of Chemistry, University of Hyderabad, Hyderabad 500046, India. Correspondence and requests for materials should be addressed to J.W.Z. (email: jwzhang@simm.ac.cn)

**Supplementary Information**

**Note. 1 Grey value analysis**

After the reconstruction based on the filtered back-projection algorithm and phase contrast extraction with X-TRACT, the reconstructed slice stack was converted into 8 bit grayscale format and re-sliced vertically. Then the vertical slices of samples were analyzed to determine the threshold gray values to distinguish the excipients, clopidogrel particles and capsule shell (Fig. S1).


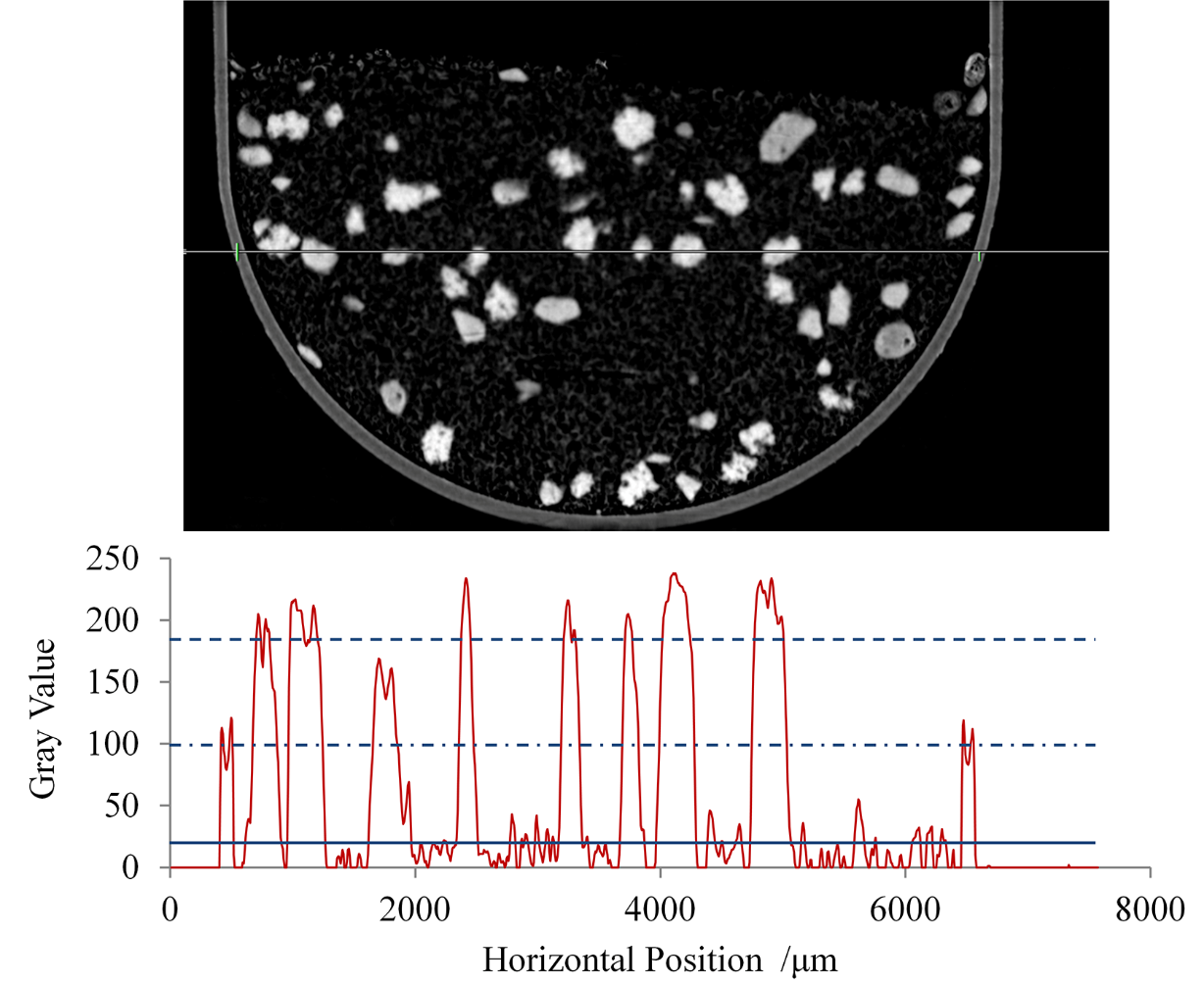


**Figure S1∣Line profile of vertical slice of sample in the capsule** (after background correction and noise reduction the gray value of background is near 0, the line at 25 shows the gray value of excipients (in this sample PVP/VA), the line at the value of 100 is the gray value of the capsule film, the line indicates that the average gray value of clopidogrel crystal is 185).

As shown in Fig. S1, clopidogrel can be distinguished from the excipients and most of the clopidogrel crystal particles were separated by diluents. Owing to the successful noise reduction and phase retrieval procedures, the gray value of background is close to 0, the excipients have a gray value of 50, the gray value between 80 and 120 is the shell of the capsule, and the clopidogrel particles have the gray value above 150.

**Note. 2 Definition of important quantitative parameters**

In order to characterize the morphological information of particles, more than thirty parameters were calculated, most important ones as follow:

**Size**: *could be derived from volume, surface area*.

**Equivalent diameter**: *is defined as the diameter of a ball of equivalent volume. Volume fraction: Ratio of object's volume to the bounding box volume (R = Vobj / Vbox)*.

**Sphericity**: *6 volumes of object divided by equivalent diameter and surface area of object. For a spherical object this parameter equals 1, for all other shapes it is less than 1*.

**Surface deviation**: *The deviation of endpoints of triangle normal vectors. Uniform surface will have deviation of 0. The maximum deviation of 1.336 will have a sphere*.

**Radius Min**: *Minimum distance between an object's centroid and surface*.

**Radius Max**: *Maximum distance between an object's centroid and surface*.

**Radius Ratio**: *Ratio between Radius (max) and Radius (min)*.

**Feret Min**: *Minimum distance between two parallel planes enclosing an object*.

**Feret Max**: *Maximum distance between two parallel planes enclosing an object*.

**Feret Ratio**: *Ratio between Feret (max) and Feret (min)*.

**Box ratio**: *Ratio between maximum and minimum size of the bounding box (R = Max / Min)*.

**Note. 3 Validation of the quantitative polymorph identification methods based on VBP**

As all the microcrystalline particles have been scanned twice in pure and mixed samples, and a quantitative method has been devised and introduced to recognize particles in polymorph mixture, it is possible to track each individual particle in different scanned samples. For the sample containing the mixed particles of CLP I and CLP II, after the CT scan, reconstruction and 3D model construction, particles in the sample have been extracted and quantitative parameters calculated. Subsequently particles were analyzed one by one to classify the crystal form with the VBP value. Then for each particle with classified crystal form, a matching process has been performed on particles in corresponding individual sample of CLP I or CLP II with the lower proportion in the mixture to find the matched particle based on size related and morphological parameters such as volume, surface area, diameter, sphericity and radius min (Fig. S2).


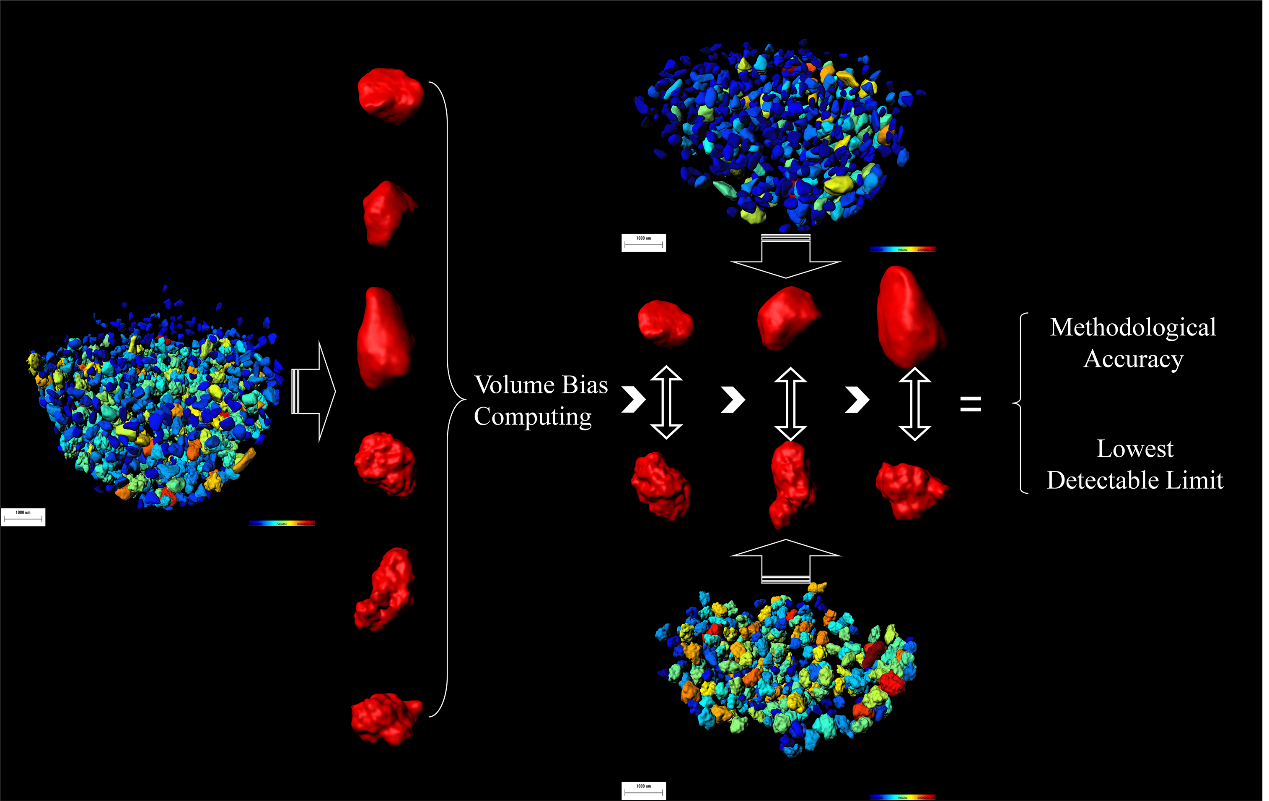


**Figure S2∣One by one particle identification and matching.**

For the validation, 4544 particles have been analyzed and the volume, surface area and sphericity selected as key parameters for matching (Fig. S3). The results are shown in Table S1. In total 4540 particles were matched. The matching rate was 99.91%.


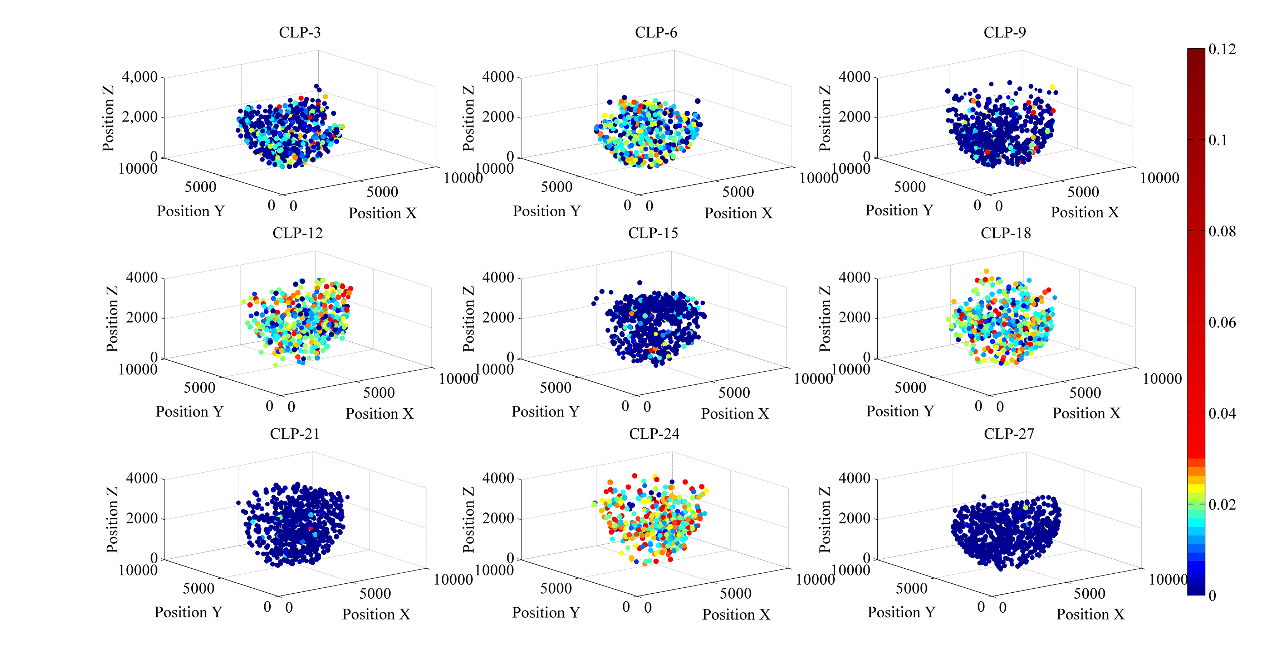


**Figure S3∣VBP value based in situ crystal form identification for polymorphic mixture (n = 4544).**

**Table S1∣**Accuracy and lowest detectable limit.

| **Sample ID** | **Content (mg)** | | **Identified** | | **Matched** | | **Accuracy %** |
| --- | --- | --- | --- | --- | --- | --- | --- |
|  | CLP I | CLP II | CLP I | CLP II | CLP I | CLP II |  |
| CLP-3 | 2.50 | 2.50 | 297 | 202 | 297 | 202 | 100.00 |
| CLP-6 | 1.00 | 4.00 | 99 | 313 | 99 | 313 | 100.00 |
| CLP-9 | 4.00 | 1.00 | 498 | 68 | 497 | 69 | 99.64 |
| CLP-12 | 0.50 | 4.50 | 48 | 380 | 49 | 379 | 99.53 |
| CLP-15 | 4.50 | 0.50 | 518 | 47 | 518 | 47 | 100.00 |
| CLP-18 | 0.25 | 4.75 | 30 | 413 | 31 | 412 | 99.54 |
| CLP-21 | 4.75 | 0.25 | 576 | 17 | 575 | 18 | 99.71 |
| CLP-24 | 0.05 | 4.95 | 9 | 390 | 9 | 390 | 100.00 |
| CLP-27 | 4.95 | 0.05 | 635 | 4 | 635 | 4 | 100.00 |
